# Examining Ownership Equity as a Psychological Factor on Tourism Business Failure Forecasting

**DOI:** 10.3389/fpsyg.2019.03048

**Published:** 2020-01-29

**Authors:** Tomasz Korol, Anastasia Spyridou

**Affiliations:** Faculty of Management and Economics, Gdańsk University of Technology, Gdańsk, Poland

**Keywords:** business failure, tourism and hospitality, psychological factors, stepwise weight assessment ratio analysis, equity

## Abstract

This paper examines ownership equity as a predictor of future business failure within the tourism and hospitality sectors. The main goals of this study were to examine which ratios are the most important for a tourism business failure forecasting model and how significant is the “total percentage of equity ownership by company directors” ratio compared with other ratios associated with the probability of bankruptcy. A stepwise weight assessment ratio analysis (SWARA) was applied, and 12 tourism bankruptcy experts evaluated key ratios. Total percentage of equity ownership by company directors is considered a psychological factor, and it was identified as the fourth most important ratio for a business failure forecasting model. Academicians and practitioners can use the findings of this study whenever developing a forecasting model for tourism and hospitality enterprises.

## Introduction

Thomas Cook, one of the most well-known tourism companies in the world, suddenly declared bankrupt on September 23, 2019. The namesake and founder of the company, Thomas Cook, was a pioneer in the tourism/travel industry. He was the first person to systematically plan, organize, and personally host railway trips; most famously, his first railway excursion in 1841 accommodated about 500 passengers with inexpensive roundtrip tickets. There are several factors that might help explain and better understand this shocking bankruptcy.

The scholars Law et al. (2004) suggested at least three possible factors: (1) uncertainty and unpredictability associated with change stemming from Brexit (the United Kingdom’s exit from the European Union), (2) the inability to cope with the increased/integral use of the internet across the travel industry, and (3) the failure of managers to develop new business models and/or adapt to emerging trends in various tourism industries. These are factors that contributed to the bankruptcy of Thomas Cook, but these are plausible factors that can/do impact industries of all kinds.

Some researchers have investigated the predictability and/or relative likelihood of a bankruptcy. For instance, Kim et al. (2012) have proposed contingency plans to mitigate business failures by using different financial indicators. Businesses in the hospitality and tourism industries are highly exposed to numbers of risks, yet very little interest has been shown in how to foresee a possible bankruptcy (Jang and Park, 2011). Predicting the likelihood of bankruptcy within the hospitality and tourism sectors is critical, especially given the unique determinants of a systematic risk associated with the characteristics and complexity of these sectors (Lee et al., 2015).

Although businesses are commonly interconnected via financial issues and concerns (Fotiadis et al., 2019b), all business enterprises are also inescapably affected by their owners (Hodari et al., 2017). Theories have examined businesses inclusive of both financial and human aspects (Smith et al., 2016; Fotiadis et al., 2019a). For example, Thomas Cook and Sons (aka Thomas Cook) was continued by his family, thus ensuring that his imprint was noticeable even as they expanded the company. Some studies indicate that entrepreneurs such as Thomas Cook are uniquely different and have a significant impact on the strategic direction and a business’s competitive advantage. However, it is extremely difficult to explore in-depth the nuances and subtleties of every entrepreneur, founder, owner, or especially the often-changing chief executive officers (CEOs) of a company. Alternative ways of predicting bankruptcy are recommended, ways that draw upon commonalities of all business enterprises. One way lies in viewing financial information found on companies’ balance sheets from a psychological point of view.

One generally accepted psychological financial factor is ownership equity. Ownership equity is well supported by agency theory. Ownership control agents, such as provision incentives in the form of equity to managers, transform managers not only into owners but into value maximizers according to Valenti and Schneider (2012). Yet there is a gap in this literature that raises a question, “How can ownership equity be used as a psychological factor in order to predict future business failure (bankruptcy) within the tourism and hospitality sectors?” To answer this guiding research question, there are two key sub-questions: which ratios are the most important for a tourism business failure model and how significant is “total percentage of equity ownership by company directors” ratio compared with other ratios associated with the probability of bankruptcy?

Vast amount of literature focuses on developing the bankruptcy forecasting model using various statistical or artificial intelligence methods; and regardless of the forecasting technique (linear or non-linear, regression or classification), majority of developed models are based solely on the use of financial ratios of analyzed companies alone. These models lack the connection between corporate finance and behavioral management. In the literature, no attempts have been made to verify the influence of any psychological factors on the corporate bankruptcy risk. The question arises whether psychological factors can be relevant predictors of a company’s risk of bankruptcy. The contribution of the paper is threefold. First, it investigates the best predictors of the financial crisis in the enterprise. Second, it determines the importance of psychological factor such as total percentage of equity ownership by company directors on the risk of bankruptcy in the company. Third, it identifies the need of implementation of bankruptcy risk forecasting models (with the use of both financial and psychological variables) in the tourism and hospitality sector.

### Business Failure

Business failure is commonplace in today’s dynamic global business ecosystems. Business failure generally puts entrepreneurs in an extremely uncomfortable position as their companies cannot generate enough profit to pay their reliabilities or generate enough cash flow to meet their operating expenses. Such an uncomfortable situation can ultimately force companies to file for bankruptcy, leading to layoffs, liquidation of existing assets, and ultimately total discontinuation of business operations. As shown in Table 1, there are several definitions attributed to business failure. The commonality among the definitions provided in Table 1 refers to business failure as the stage when a company has to cease its operations owing to the inability to survive in the industry.

**TABLE 1:** Business failure definitions.

Definition	Study
Entrepreneurial business failure refers to the inability of the entrepreneurs (managers) to mobilize expertise and resources necessary to mitigate weaknesses and threats, resulting in the collapse of an entrepreneurship	Zhang et al., 2019
Business failure refers to the stage where a company has to stop all business operations or go out of business	Amankwah-Amoah, 2016
Business failure refers to voluntary shutting down of business operations owing to poor performance, resulting in insolvency, liquidation, or closure	Garcia Martinez et al., 2019
Business failure can be viewed as a synonym of “distressed businesses” and collapses of businesses. However, some “distressed businesses” can eventually transition to become collapsed businesses	Amankwah-Amoah and Wang, 2019
Business failure can be defined as the cessation of contributions to a business venture owing to its failure to meet the minimum threshold for economic viability as expected by the entrepreneur	Ucbasaran et al., 2012

Whenever entrepreneurs fail, they have to grapple with several negative psychological emotions (e.g., shame, humiliation, anger, and guilt). These states/conditions have been widely studied within the realm of cognitive dissonance theory. Cognitive dissonance, in principle, suggests that people tend to avoid the discomfort associated with difficult situations (Tang, 2014). This is analogous to a business failure. A company facing the challenges of a highly competitive business environment may force an owner to avoid competitive realities until he/she can fully understand the situation, but lamentably, this is often to no avail. Owners and their businesses might ultimately succumb to failure and bankruptcy and the emotional fallout mentioned above.

Bankruptcy of enterprises is a dynamic system, covering many endogenous factors (e.g., age and size of company, type of industry, profitability, level of indebtedness, skills of managers, the level of entrepreneurship, knowledge, and competence) and exogenous factors (e.g., business cycle, bankruptcy law, availability of capital, suppliers, customers, inflation rate, fluctuation of exchange rates, interest rates, and technology) (Shin and Song, 2012). However, those issues have not been combined into one compact, comprehensive theory. Bankruptcy of enterprises does not constitute a central element of any of the known trends of economic theory. There are only some elements of theory of bankruptcy present in selected economic theories (Table 2). This is a result of the enormous diversity of causes of bankruptcies.

**TABLE 2:** Elements of theory of bankruptcy present in selected economic theories.

Economic theory	Elements of bankruptcy theory
A. Marshall’s neoclassical theory	Bankruptcy of firms is a consequence of the withdrawal from the objective of maximizing profits
J. Schumpeter’s entrepreneurship theory	Bankruptcy of inefficient and non-innovative companies is a prerequisite for the development of the economy as a whole. For this reason, bankruptcy is beneficial for the economy
Institutional trends	The scale and pace of bankruptcy procedures in the economy are conditioned by the quality of institutional infrastructure for bankruptcy. Attention is, however, paid to the fact that bankruptcy, owing to the existence of transaction costs and agency problems, can have negative effects at the microscale and macroscale
Managerial theories	Avoiding bankruptcy is a prerequisite for achieving the objectives which managers seek. Bankruptcy excludes benefits of managers. It is also bad for the owners and partners connected with the company
Biological theories	Bankruptcy is a natural element of the company’s life cycle
Theory of value for shareholders	The desire to maximize value for shareholders ensures the survival of businesses in the long run. Bankruptcy precludes realization by the company of the postulate to maximize the value; it is therefore bad for the owners

There is a much higher entrepreneurial risk within the tourism industry than other industries. Power et al. (2017) suggested a higher likelihood of business failure that necessitates sustaining strategies in order to avoid such failure. This explains why development that sustains business strategies and the life cycle of a company, as well as ownership, are significant success factors for the tourism and hospitality sector (Getz and Carlsen, 2005; Wang et al., 2019).

### Business Failure Models in Tourism and Hospitality Sectors

There are many indicators that can help predict the extent to which a business is moving toward a red-zone failure. The extant literature offers different viewpoints and perspectives about forecasting that help identify the warning signs of corporate failure.

The use of forecasting in predicting future events is considered across numerous disciplines. For example, meteorological forecasting of dangerous weather conditions can enable individuals, communities, and governments to prepare a timely response plan that, in turn, can help mitigate any adverse physical safety and/or property impact (Ebi, 2007). Forecasting is an important tool within the tourism industry for decision making and preparations, such as projecting the number of tourist arrivals during peak and off-peak seasons. Similarly, forecasting models can be used to prepare contingency plans for tourism organizations. Financial risks are always a prime factor that can determine business success or failure. Consequently, it is essential for a business to develop a financial warning system that uses reasonable forecasting models. When doing so, business leaders can anticipate vulnerabilities and thereby prepare backup plans (Bucevska, 2011; Nik et al., 2016). Clearly, the using of forecasting models is important for strategic, even emergency, decision making. According to Kaur (2015), forecasting models are tools that decision makers can use to assess weaknesses and vulnerabilities in order to minimize and/or buffer any risks to such exposure.

However, forecasting models are unable to predict vulnerabilities with high-level accuracy because of the complexity of business ecosystems; it is impossible to identify all the variables linked to any kind of a market risk because dynamic future events are by nature highly emergent. As Christofides et al. (2016) pointed out, none of the well-established forecasting models were able to predict the Greece financial crisis in 2008. Nonetheless, it is important to continually improve forecasting models so that they can more accurately predict a pending or certain crisis on the horizon (Ionela, 2014, p. 166).

Currently, there are different forecasting models that can be used (Zigraiova and Jakubik, 2015; Kimmel et al., 2016) depending on where a company is situated on the spectrum of a possible financial failure. For instance, on the basis of the paradigm of business failure, Inmaculada (2017) categorized companies as chronic failure companies, a revenue financing failure company, or an acute failure company. Additionally, the literature offers several forecasting models [early warning systems (EWSs)] that help identify a financial risk such as Meyer and Pifer (1970), who employed a logit model to build an EWS for the banking sector. Another example is Inmaculada (2017), who used the Cox regression model to explore the nexus between the risk of failure and different types of positioning for a company. And Korol (2013) used a variant of the artificial intelligence model to predict bankruptcy of enterprises across Latin America and Central Europe with discriminant analysis, decisional trees, and artificial neural networks (ANNs). Lastly, the method fuzzy logic (FL) is one of the more sophisticated models that have been employed to predict enterprise bankruptcy (Korol, 2012).

Furthermore, the current literature includes statistic-based models and artificial intelligence-based models for forecasting enterprise bankruptcy. Alaka et al. (2018) conducted a literature review and found multiple discriminant analysis (MDA) and logistic regression (LR) as the two main statistical methods used to predict a bankruptcy. The artificial intelligence-based models such as support vector machines (SVMs), ANN, rough sets (RS), genetic algorithm (GA), case-based reasoning (CBR), and decision tree (DT) were found to be the most used techniques to forecast bankruptcy (Alaka et al., 2018; Shin and Bartolacci, 2007). Financial ratios (e.g., liquidity and profitability) are the most commonly used indicators to forecast business performance and bankruptcy (Thai Siew and Abdollahi, 2013). All the abovementioned forecasting methods use different indicators to estimate performance or bankruptcy and are invaluable to owners and strategic leaders in the hospitality and tourism industries.

Using logit and discriminant analysis, Pereira et al. (2017) employed an econometric model and a multivariate model to predict the likelihood of business failure in the hospitality sector in Portugal. To this end, the authors used a historical dataset spanning from 2009 to 2013 that included a sample of 230 Portuguese companies. These researchers calculated 30 different financial ratios that were based on the balance sheets and income statements of those 230 companies. The most important ratios were current assets/short-term liabilities; cash flow/total liabilities; cash/current liabilities; working capital/total assets; and operating profit/operating costs. On the basis of the results of their study, Pereira et al. (2017) claimed that forecasting business failure might enable policymakers to design macroeconomic policies and tourism development programs accordingly.

Barreda et al. (2016) investigated the likelihood of corporate failure in four categories of business in the hospitality sector in the United States, namely, restaurants, hotels, resorts, and casinos. In their study, the authors used the logit model and MDA to determine which of these two models provide the more accurate forecast on a dataset spanning from 1992 to 2010. The key financial indicators used in their study were return on asset (ROA), quick ratio, debt equity, and asset turnover. Barreda et al. (2016) found the MDA model outperformed the logit model for overall bankruptcy forecasting.

Gémar et al. (2016) examined the likelihood of survival in the Spanish hotel industry using a sample of 1,033 hotels spanning from 1997 to 2009. Besides the financial indicators, they also included non-financial indicators in their analysis (e.g., size, location, type of hotel, management, and the launch time). They used an econometric analysis of survival, specifically the non-parametric Kaplan–Meier estimator of constructed variables to assess the influence of each variable. Furthermore, they used the Cox proportional hazards model to assess which variables influence the survival of hotels. The results of Gémar et al. (2016) study indicated that indeed the survival of firms in the hotel sector depends primarily on four factors: the size, location, management, and launch time during a period of prosperity. Lado-Sestayo et al. (2016) also investigated the determinants of survival within the Spanish hotel sector using a sample of 6,494 hotels located in 97 different tourist destinations and an impressive dataset spanning from 2005 to 2011. Lado-Sestayo et al. (2016) found that location was a significant determinant that affects the probability of survival of the hotels in their study. They also found that a low level of competition decreases the probability of expected survival. The results of their study indicated a positive relationship between the average profitability of a tourist destination and the firm’s ultimate survival.

In a more recent study, Gemar (2019) examined factors that influence resort hotel survival in Spain. The author employed Cox’s semi-parametric proportional hazards regression to examine which factors influence hotel bankruptcy and by how much each factor increases the risk of bankruptcy. Gemar (2019) found that bankruptcy in the case of resort hotels in Spain depends on the size, location, business cycle, executive management, and the business cycle; bankruptcy did not depend upon hotel type or financial structure.

Kim and Gu (2006) developed a logit model and compared it to a discriminant model in order to determine which model provides a more accurate forecasting of bankruptcy. They found that both models actually provide an accuracy rate of 94% for in-sample restaurant businesses and 93% accuracy for the out-of-sample businesses 1 year prior to bankruptcy. However, Kim and Gu (2006) recommended the use of a logit model versus the discriminant model because of its theoretical soundness. Park and Hancer (2012) compared the logit model and ANNs for forecasting bankruptcy within the hospitality industry. The results of their study showed that the ANN provides a higher accuracy rate for the in-sample test than does the logit model. Park and Hancer (2012) also found that the logit and ANN models both achieved a 100% accuracy rate with a holdout sample.

Several researchers have tried to identify the main factors associated with the likelihood that a company will file for bankruptcy within a few years. Shkurti and Duraj (2010) considered Beaver (1967) as the pioneer of statistical methodologies for predicting bankruptcy by using a univariate approach. In a later study, Altman (1968) introduced the use of five financial ratios in a multifactor analysis (discriminant analysis) to test the financial forecasting model for predicting the likelihood of bankruptcy and non-bankruptcy among a number of trading companies across industries and countries. To date, this method and study have been the most impactful for forecasting firm bankruptcy. Specifically, Altman (1968) provided initial financial forecasting, as follows: *Z*-score = 1.2(*A*) + 1.4(*B*) + 3.3(*C*) + 0.6(*D*) + 1.0(*E*), where *A* represents the working capital/total ratio; *B* the retained earnings/total assets ratio; *C* the earnings before interests and taxes/total assets ratio; *D* the market value of equity/book value of total liabilities; and *E* the sales/total assets ratio. Another vital consideration is the inverse relationship between time horizon and forecasting accuracy. Lin et al. (2014) have shown that in most cases an increase in time horizon is likely to decrease forecasting accuracy.

Nowadays, there persists a continued and growing argument over the most appropriate forecasting models. In an earlier study, Ohlson (1980) argued that MDA is not the most accurate forecasting model and suggested the use of the LR (logit) model developed by Cox (1958) as a better alternative. Mihalovič (2016) compared the MDA model and the logit model to assess which one of these two models better predicts bankruptcy in the Slovak Republic and found that the logit method outperformed the MDA model. Garcia-Gallego et al. (2015) similarly determined that the logit model indeed outperformed the MDA model.

The probit method is a statistical method that is comparable with the logit method and is often used in forecasting analysis. Similar to the logit models, the probit models uses a dichotomous binary variable. However, the probit model differs from the logit model because it assumes that variables are normally distributed (Klieštik et al., 2015). The probit model has also been compared with other models. For example, MDA, logit, probit, and ANN models were used to examine bankruptcy and non-bankruptcy of public firms in Taiwan using a dataset that spans from 1998 to 2005. The authors found that the probit, logit, and ANN models achieved the highest prediction accuracy of these models (Klieštik et al., 2015).

There has been a sustained growing interest in recent years for using soft computing methodologies to predict bankruptcy. Although soft computing methodologies might seem to provide more accurate predictions than do statistical methods, their sophisticated makeup and prerequisite specific knowledge and skill sets pose substantial difficulty for implementation and widespread usage. Among the most sophisticated soft computing methods used in forecasting are self-organizing maps (SOMs), multilayer perceptron (MLP), learning vector quantization (LVQ), radial basis function (RBF) networks, relevance vector machines (RVMs), and SVMs (Ribeiro et al., 2016). Also, the most applied forecasting methodologies according to Verikas et al. (2010), p. 995) are hybrid systems such as probabilistic neural networks (PNNs), Bayesian networks (BNs), DTs, GA, CBR, fuzzy DTs (FDTs), FL, and RS.

### Importance of Ownership Equity

Informational and agency issues are two important factors that are associated with ownership structure and financial performance of firms. Mykhayliv and Zauner (2017) suggested these as important motives to establish an equity position. In principle, corporate equity positions can help mitigate contracting problems between firms during the process of a joint venture or alliance formation. Farrell and Shapiro (1990) investigated conditions under which a firm with an initial equity position would likely increase its equity stake. In the context of perfect capital markets, Farrell and Shapiro argued that an increase in size of an equity stake leads to two different effects on firm profitability. When the firm has a large stake in the rival company, it will show greater interest in its profitability. In this case, the firm may be tempted to lower the size of its equity so that the rival can earn a much larger profit. In turn, this will lead to an increase in the value of the firm’s initial equity stake but inversely result in a decrease in the firm’s own operating profits. Farrell and Shapiro (1990) therefore concluded that a firm tends to increase its equity position only if the increase in the value of the initial equity stake surpasses the decrease in operating profits.

## Methodology

In this study, we posit that the percentage of equity owned by the director of a company can function as a psychological factor that might determine the likelihood of failure/bankruptcy of a tourism business. To test this assumption, we applied an expert judgment method using the following four steps. First, we selected and confirmed the variables to include in the expert judgment method; this study utilized key financial ratios as key variables. Second, we created a list of 16 specific financial ratios to be analyzed. Third, 12 experts were enlisted who ultimately confirmed their participation in this study. Lastly, the experts were asked to rate the importance of each variable.

After completing these four steps, we employed the stepwise weight assessment ratio analysis (SWARA) method as suggested by Hashemkhani Zolfani et al. (2018). The use of the SWARA method is supported for its reliability in assessing experts’ opinions related to the rate values and weight values of criteria *i* and its usefulness in supporting the coordination and gathering the data from consulting experts (Hashemkhani Zolfani et al., 2018). The SWARA method is subjective criteria-weighting that is widely used in the fields of management, economics, management, policy and environmental sustainability industry, manufacturing, and design and architecture (Ghenai et al., 2020). The steps employed for the SWARA method were as follows.

First, it is important to calculate the values of *t*_*jk*_. The average attribute value of $\bar{t_{j}}$ is obtained using the following formula:

$$\bar{t_{j}}=\frac{\sum_{k=1}^{r}t_{j\,k}}{r}$$

where *t*_*jk*_ represents the ranking of the *j* attribute by the *k* respondent, and *r* is the number of respondents.

Second, it is necessary to identify weights *q*_*j*_. The weights of the attributes are thus calculated by dividing the mean value of each attribute by the sum of the attributes priority values (*t*_*j*_) by

$$q_{j}=\frac{\bar{t_{j}}}{\sum_{j=1}^{n}t_{j}}$$

and the variation of the obtained values can be detected using the following formula:

$$\beta_{j}=\frac{\sigma }{{\bar{t}}_{j}}$$

Third, the weighted values should be calculated. We can determine the reliability of the data by the coefficient of concordance in the responses provided by experts. In the case of repeated rankings for the same variables, as in our case, the coefficient of concordance is obtained using the following formula:

$$W=\frac{12\,S}{r^{2}\,\left(n^{3}-n\right)-\sum_{k=1}^{r}t_{k}}$$

where *S* is the total square deviation of the rankings of each attribute; *t*_*k*_ the index of the repeated ranks in the *r* rank; *r* the number of respondents; and *n* the number of evaluation attributes.

Fourth, the values of χ^2^ are calculated and obtained using the following formula:

$$X_{a,v}^{2}=W.r.\left(n-1\right)=\frac{12\,S}{r.n\,\left(n+1\right)-\frac{1}{n-1}=\sum_{k=1}^{r}t_{k}}$$

In testing the χ^2^ > χ^2^, when the calculated value χ^2^ is superior to the critical tabular value χ^2^ for the selected level of significance (e.g., α = 0.05), then the hypothesis regarding the concordance agreements of the experts cannot be rejected. Also, when χ^2^α, *v* > χ^2^, the significance of concordance coefficient exists on the α level; therefore, we can conclude that group opinion is established, meaning that the experts have the same opinions. On the basis of the second criterion, the respondent assesses the relative importance of criterion *j* based on the previous (*j* - 1) criterion, for each specific criterion. And the coefficient *k*_*j*_ can be obtained using the following equations:

$$k_{j}=\left\{ \begin{array}{c} 1,a\,n\,d\,j=1\\ s_{j}+1,a\,n\,d\,j>1 \end{array}\right.$$

$$q_{j}=\left\{ \begin{array}{c} 1,a\,n\,d\,j=1\\ \frac{k_{j}-1}{k_{j}},a\,n\,d\,j>1 \end{array}\right.$$

Finally, the relative weights of the evaluation criteria can be obtained using the following equation:

$$w_{j}=\frac{q_{j}}{\sum_{j=1}^{n}q_{j}}$$

where *w*_*j*_ denotes the relative weight of criterion *j*.

## Results

In the section “Methodology,” the need to identify the ratios was established and thus included in the analysis required by the SWARA method. In our study, a list of 30 ratios was provided to three experts who selected the ratios that they deemed are most suitable in forecasting business failure within the tourism and hospitality sectors. Table 3 presents the most reliable ratios based on these experts’ judgment.

**TABLE 3:** Business failure ratios.

Name	Definition
WCTA	Working capital/total assets
RETA	Retained earnings/total assets
EBITTA	Earnings before interest and taxes/total assets
TLTA	Total liabilities/total assets
METD	Market value of equity/total debt
STA	Sales/total assets
XRD	The ratio of research and development expenses to sales
NITA	Net income/total assets
MAN	The total percentage of equity ownership by company directors
	

After the selection of the most appropriate ratios by the first three experts, the list of ratios was then given to 12 additional experts. The 12 experts were tourism-related academicians with significant experience in finance and publication records (see Table 4).

**TABLE 4:** Ratio significance as indicated by experts.

	XRD	NITA	METD	MAN	EBITTA	STA	WCTA	RETA	TLTA
Ex1	1	2	3	5	7	6	9	8	4
Ex2	1	3	2	7	8	6	9	4	5
Ex3	1	2	3	4	5	7	9	8	6
Ex4	2	1	6	8	7	3	9	5	4
Ex5	1	3	2	7	8	6	8	4	5
Ex6	2	1	3	4	6	7	9	8	5
Ex7	1	4	2	6	9	3	7	8	5
Ex8	1	2	3	7	5	4	9	8	6
Ex9	1	2	3	7	4	6	8	9	5
Ex10	2	4	1	3	5	8	9	7	6
Ex11	1	2	3	5	9	6	7	8	4
Ex12	3	1	2	5	6	4	7	9	8

The experts gave nine points for the ratios considered the most important and 1 point for the ratio they considered the least important.

The SWARA methodology has several progressive steps. As Table 5 indicates, the sum of ranks was elaborated and indicated that the most significant ratio is WCTA followed by RETA. The MAN ratio, which relates to ownership equity, is ranked as the fourth most significant, thus demonstrating the importance of this psychological ratio.

**TABLE 5:** Stepwise weight assessment ratio analysis (SWARA) methodology.

	XRD	NITA	METD	MAN	EBITTA	STA	WCTA	RETA	TLTA
Sum of ranks	17.00	27.00	33.00	68.00	79.00	66.00	105.00	86.00	63.00
Avg. attribute rank value	1.42	2.25	2.75	5.70	6.60	5.50	8.30	7.17	5.25
Attribute rank	9	8	7	4	3	5	1	2	6
Attribute weight	0.03	0.05	0.06	0.13	0.15	0.12	0.19	0.16	0.12
Dispersion	0.45	1.11	1.48	2.42	2.81	2.64	0.79	3.24	1.30
Variation	0.47	0.47	0.44	0.27	0.25	0.30	0.11	0.25	0.22
Ranking sum average *V*	60	=45*12/9							
Total square ranging deviator	1,849	1,089	729	64	361	36	2,025	676	9
Coefficient (*W*)	0.79	=12*6,838/[12^2*(9^3–9)]							
Significance of the concordance coefficient	75.98	=12*6,838/(12*9*10)							
Rank of table concordance	The null hypothesis H_0_: The consent of experts in rankings is not accepted. Degrees of freedom (df) are *v* = 9 - 1 = 8, which is # of categories minus 1. The indication is 75.97 > 15.5 at α = 5%								
Compatibility of expert judgment	Hence, we can reject the null hypothesis H_0_.								

**FIGURE 1:**
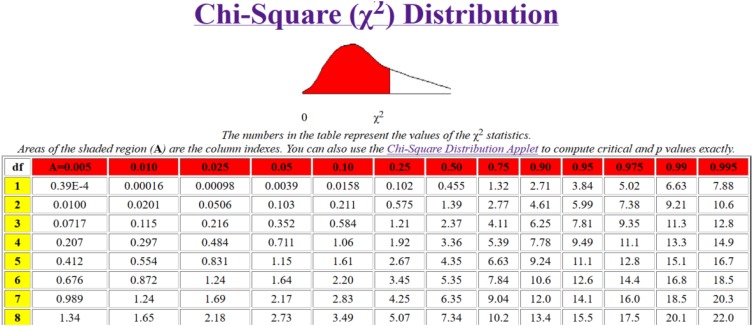
Chi-square (χ^2^) distribution.

Hypothesis H_0_ was established. *H_0_: The consent of expert ranking is not accepted*. On the basis of the results shown in Table 5, we can see that the degrees of freedom are 8 for the rank of table concordance. Because the significance of concordance coefficient is higher than 15.5, the null hypothesis is rejected. The rejection of the null hypothesis signifies that the experts’ consent of rankings is accepted. This can also be viewed in Figure 1.

As can be seen, the calculated χ^2^ value is 75.97. The tabular value of χ^2^ is at a 5% significance level with 8 degrees of freedom of 15.5. Thus, the alternative hypothesis that “the consent of experts in rankings is accepted” is found to be valid.

Evaluation of the final weighting was the next and final step following the SWARA methodology (Table 6). This step utilizes the average attribute rank values and comparative importance values and requires dividing the higher category by the category above. For example, WCTA/RETA = 7.17/8.30 = 0.86. The new comparative value is added to the coefficient. Then, to arrive at a recalculated weight, the previous category recalculated weight should be divided by the new coefficient. In this case, the RETA ratio will be 1.00/1.86 = 0.54. Finally, the new weight can be found by first finding the sum of the recalculated weight and then dividing each weight by the sum. In this instance, the sum equals to 2.13. So in the case of WCTA, it should be 1.00/2.13 = 0.47. Again, the new recalculated weight indicates that WCTA is the most important ratio and MAN is still remains in the fourth position rank. Lastly, the least important ratio is XRD with a 0.00 importance weight, which means that this ratio can be excluded from a future business failure forecasting model.

**TABLE 6:** Stepwise weight assessment ratio analysis (SWARA) model: final weighting.

Criteria	Average attribute rank values	Comparative importance values	Coefficient	Recalculated weight	Weight
WCTA	8.30	–	1.00	1.00	0.47
RETA	7.17	0.86	1.86	0.54	0.25
EBITTA	6.60	0.92	1.92	0.28	0.13
MAN	5.70	0.86	1.86	0.15	0.07
STA	5.50	0.96	1.96	0.08	0.04
TLTA	5.25	0.95	1.95	0.04	0.02
METD	2.75	0.52	1.52	0.03	0.01
NITA	2.25	0.82	1.82	0.01	0.01
XRD	1.42	0.63	1.63	0.01	0.00

## Conclusion

In the business failure forecasting literature, one of the biggest problems is determining which ratios should be used for the development of a dependable forecasting model. Typically, previously used ratios were strictly economic ones. However, during more recent research, other types of ratios have been used in the attempt to bolster this approach in order to increase the success in predicting future bankruptcies/failures. In particular, a psychological ratio related to economic ratios was evaluated for its importance and was tested.

There are different ways to evaluate the significance of ratios, and in this study, a SWARA method was considered the most suitable. The SWARA method is a relatively new, subjective criteria-weighting method commonly used in the fields of economics, manufacturing, industry, management, design, and architecture, as well as in developing environmental sustainability policies. The SWARA method, in contrast to other weighting methods, is an uncomplicated and straightforward method that uses a few select numbers of comparisons. Most importantly, the SWARA method has outperformed the other weighting methods when addressing which factor or process to prioritize within given economic, environmental, and policy conditions.

As the results in this study show, experts’ judgment was considered significant; and the MAN ratio, the total percentage of equity ownership by company directors, was the fourth most important ratio for the development of a business model. Although highly promising, continued studies should be developed to further explore how psychological factors inside or outside a company could likely affect possible futures for tourism and hospitality business failure or success.

The authors are aware of various limitations of the conducted study. The main difficulty is limited access to the information on the total percentage of equity ownership by company directors in the bankrupt enterprises. The ideal situation would be a possibility to develop the forecasting model with the use of such psychological variable separately for small- and medium-sized enterprises and large firms.

Nevertheless, the authors of this research are going to continue the study by adding this psychological factor as a non-financial variable to the early warning model. Knowing that such factor can play important role in the assessment of a bankruptcy risk, we can try to implement it to the model with other commonly used financial ratios.

## Data Availability Statement

All datasets generated for this study are included in the article/supplementary material.

## Ethics Statement

Ethical review and approval was not required for the study on human participants in accordance with the local legislation and institutional requirements. The patients/participants provided their written informed consent to participate in this study.

## Author Contributions

All authors listed have made a substantial, direct and intellectual contribution to the work, and approved it for publication.

## Conflict of Interest

The authors declare that the research was conducted in the absence of any commercial or financial relationships that could be construed as a potential conflict of interest.
